# Geographic proximity to primary care providers as a risk-assessment criterion for quality performance measures

**DOI:** 10.1371/journal.pone.0273805

**Published:** 2022-09-06

**Authors:** Nathaniel Bell, Ana Lòpez-De Fede, Bo Cai, John Brooks

**Affiliations:** 1 College of Nursing, University of South Carolina, Columbia, South Carolina, United States of America; 2 Institute for Families in Society, University of South Carolina, Columbia, South Carolina, United States of America; 3 Arnold School of Public Health, University of South Carolina, Columbia, South Carolina, United States of America; Auburn University, UNITED STATES

## Abstract

**Importance:**

Previous studies have found a mixed association between Patient-Centered Medical Home (PCMH) designation and improvements in primary care quality indicators, including avoidable pediatric emergency department (ED) encounters. Whether these associations persist after accounting for the geographic locations of providers relative to where patients reside is unknown.

**Objective:**

To examine the association between geographic proximity to primary care providers versus hospitals and risk of avoidable and potentially avoidable ED visits among children with pre-existing diagnosis of attention-deficit/hyperactivity disorder or asthma.

**Methods:**

Retrospective cohort study of a panel of pediatric Medicaid claims data from the South Carolina from 2016–2018 for 2,959 beneficiaries having a pre-existing diagnosis of attention-deficit/hyperactivity disorder (ADD, ages 6–12) and 6,390 beneficiaries with asthma (MMA, ages 5–18), as defined using Healthcare Effectiveness Data and Information Set (HEDIS) performance measures. We calculated differences in avoidable and potentially avoidable ED visits by the beneficiary’s PCMH attribution type and in relation to differences in proximity to their primary care providers versus hospitals. Outcomes were defined using the New York University Emergency Department Algorithm (NYU-EDA). Differences in ED visit risk were assessed using generalized estimation equations and compared using marginal effects models.

**Results:**

The 2.4 percentage point reduction in risk of avoidable ED visits among children in the ADD cohort who attended a PCMH versus those who did not increased to 3.9 to 7.2 percentage points as relative proximity to primary care providers versus hospitals improved (p < 0.01). Children in the ADD and MMA cohorts that were enrolled in a medical home, but did not attend one for primary care services exhibited a 5.4 and 3.0 percentage point increase in avoidable ED visit compared to children who were unenrolled and did not attend medical homes (p < 0.05), but these differences were only observed when geographic proximity to hospitals was more convenient than primary care providers. Mixed findings were observed for potentially avoidable visits.

**Conclusions:**

In several health care performance evaluations, patient-centered medical homes have not been found to reduce differences in hospital utilization for conditions that are treatable in primary care settings among children with chronic illnesses. Analytical approaches that also consider geographic proximity to health care services can identify performance benefits of medical homes. Expanding risk-adjustment models to also include geographic data would benefit ongoing quality improvement initiatives.

## Introduction

Ample evidence shows that health outcomes across the United States (US) differ by race, ethnicity, and socioeconomic status [1–5]. For instance, emergency department (ED) utilization is higher among the homeless [6], diabetes prevalence is often attributed to greater food insecurity [7], and social isolation contributes to both stroke and heart disease [8], to name but a few of its determinants and consequences. It is also possible to make a reasonably good prediction as to how long it will take people to access care or the type of treatment they will receive based on knowing where they live [9–11]. For example, recent studies have shown that Medicaid beneficiaries who live closer to EDs have a higher probability of using these services for non-emergent reasons than other patient groups [12].

Inappropriate or non-emergent ED utilizations raise costs, strain resources, as well as increase wait times for care [13,14]. They are also fundamentally linked to social determinants of health, such as poor primary care access or low health literacy [15], as well as to care management on the part of patients and primary care providers [16]. Yet, despite national recommendations on the general management of chronic illnesses and the substantial cost and negative implications of non-emergent ED visits, pre-COVID 19 pandemic studies found that the frequency of avoidable visits were increasing, particularly among children [17]. For example, it is estimated that nearly 75% of the 3 million ED visits for asthma-related care and nearly 33% of the 1.6 million ED visits for mental health-related conditions can be treated in primary care settings [18–21]. Medicaid is often the most frequent payer of ED visits nationwide [22].

Over the past decade, patient-centered medical homes (PCMH) have shown potential to limit avoidable ED utilizations and improve preventative and chronic disease management [23,24]. Akin to other patient-based practice structures, such as Canada’s Family Health Teams [25], medical homes are largely based on a physician-led continuity of care model that include nurses, nutritionists, pharmacists, case managers, and social workers with an emphasis on providing patient-and culturally-centered care [26]. In the broadest sense, they represent a transformative whole-person approach to primary care designed to improve physical health, behavioral health, access to community-based social services, as well as better management of chronic illnesses [27–30]. Aspects of medical home design, such as its use of care coordinators to help with medication reconciliation are particularly relevant for children with chronic conditions who have a diverse set of medical needs [31]. For instance, case managers may work directly with children and their guardians to coordinate care, help families navigate the health care system, as well as coordinate appointments with specialists to improve disease control [32].

Although there is growing consensus of the benefit of PCMH-modeled care on health outcomes overall, including lowered ED utilization, its effectiveness in reducing pediatric ED utilizations have been mixed. Among the general pediatric population, evaluations have shown a reduction in ED use as well as improvements in preventative care delivery [33–35], but typically show little effect on usage rates among children with chronic illnesses, even when evaluated using patient-reported quality metrics (e.g., wait times to care, satisfaction with providers) [36–38]. Mixed findings are thought to stem from the amplified effect that care fragmentation and poor communication across resource systems have on children with greater medical needs [39]. It is also possible that a caregiver’s perception that their child is not receiving sufficient care may be a stronger predictor of ED use regardless of the quality of primary services the child receives. However, missing from previous evaluations is whether the interaction between where patients live and where their health care services are located modifies these associations. Although some PCMH evaluation studies have attempted to control for geography (e.g., rural versus urban clinics) [40,41], this approach ignores the spatial connectivity between patients and providers and cannot answer questions as to whether greater travel distances to care determines why the effects of some medical home innovations are often muted.

**Fig 1:**
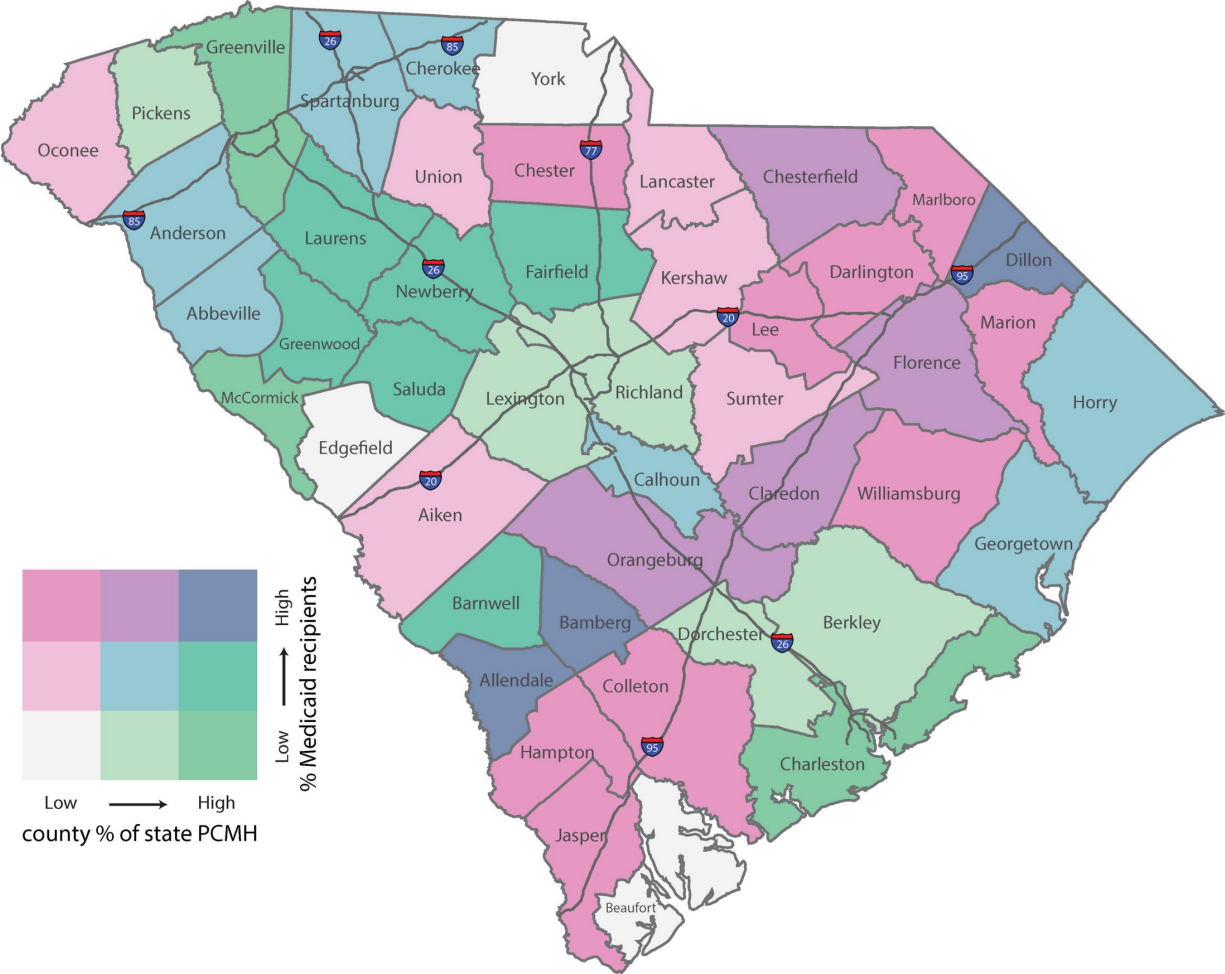
Bivariate choropleth map of county proportion of medical homes relative to all primary care providers and the proportion of county population enrolled in Medicaid. Figure created by the authors using the University of South Carolina’s site license for ArcMap. Data to recreate the map is publicly available through the American Community Survey, the NPI Certification files distributed by the Centers for Medicare and Medicaid Services, and the NCQA data feed file (NCQA data available through subscription).

In this study, we assessed whether geographic proximity to health care providers modified the association between medical home attendance and risk of avoidable and potentially avoidable ED visits among pediatric Medicaid recipients in South Carolina (SC) having a pre-existing diagnosis of asthma or attention-deficit/hyperactivity disorder, each of which are chronic illnesses that often result in ED visits for disease-specific care that is often treatable in primary care settings [42,43]. Each condition was defined using the National Committee for Quality Assurance’s (NCQA) Healthcare Effectiveness Data and Information Set (HEDIS^®^) process measures, thus reflecting a measurement standard commonly used for risk adjustment and performance tracking by state Medicaid agencies. Although many initial evaluations of the PCMH transformation on outcomes among Medicaid participants have been positive, pointing to the benefit of better compliance measures, case management, and electronic health records with earlier and better access to care [44–47], some studies have failed to show consistent improvements quality [48,49]. Fig 1 provides an example of why spatial interactions in these and other PCMH evaluations might be warranted. It shows that SC counties that have witnessed the greatest penetration of medical homes are simultaneously among the least dense with respect to the % of the population enrolled in Medicaid (areas in bright green). What is also particularly telling is the ‘I-95 corridor’, often referred to as the ‘corridor of shame’ [50], which stretches from Jasper county in the south to Dillon county in the northeast, is among the areas with the poorest access (areas in bright pink). Eight of the 17 counties that form part of the corridor are on the wrong end of the PCMH availability-to-need spectrum, many of which are among the poorest counties in the country.

Given what is known about proximity to care as a determinant of service utilization and outcomes, statistical models that include interactions with spatial data representative of where patients reside relative to where they receive primary and emergency care services may help reveal unmeasured benefits or barriers attributed to medical home-modeled care. Assessing the representation of one type of service accessibility measure (i.e., travel distances) is important for ongoing policy decisions for state Medicaid programs, which almost universally provide support, technical assistance, and resources to clinicians, practices, and families to ensure its beneficiaries gain access to designated medial homes [51]. This emphasis is also timely given the ongoing transformations of US risk-adjustment and pay-for-performance models to include social factors on top of the clinical risk factors already in place [52–54]. The NCQA is one of the most recent organizations to propose health plan accountability for social risk factors [55]. The NCQA is also one of the largest accrediting bodies that certifies primary care clinics as designated medical homes.

## Materials and methods

### Patient population

Approximately one out of every five of the 5.1 million persons residing in SC is a Medicaid beneficiary, of which 62% are 18 years of age or younger [56]. In 2013, the SC Department of Health and Human Services’ (SCDHHS) Medicaid agency began working with its managed care programs to enroll eligible beneficiaries into designated PCMHs. The intention has been to improve access to primary care, particularly care that is considered to be of higher quality. This study used a 3-year retrospective cohort design of pre-COVID 19 pandemic data, beginning with a baseline (reference) year of 2016, which is consistent with other Medicaid program effectiveness evaluations [57]. Medicaid claims were eligible for inclusion if the beneficiary was continuously enrolled for the entire 2016 reporting year, but the patient was not required to be enrolled for the entire study period. Continuous enrollment in South Carolina allows patients to churn on/off Medicaid for no more than 45 days per year.

We used the NCQA’s Healthcare Effectiveness Data and Information Set (HEDIS^®^) process measures to define a similar patient cohort and as a grouping mechanism to differentiate differences in outcomes achieved from designated and non-designated clinics. HEDIS^®^ measures comprise the core evaluation metrics for pediatric and adult Medicaid programs and relate to numerous public health issues, including cancer, heart disease, mental health, asthma, and diabetes, among others. HEDIS^®^ metrics are used to measure specific aspects of primary care quality, including the effectiveness (e.g., colorectal cancer screening) and experiences of care (e.g., CAHPS surveys), access/availability of care (e.g., access to ambulatory health services), risk adjusted utilizations (e.g., well-child visits), as well as relative resource use (e.g., resource use for people with COPD). In 2016, 37 state Medicaid programs collected or required its Managed Care plans to report on over 60 HEDIS^®^ measures as part of annual external quality reviews [58]. The use of HEDIS^®^ metrics as a grouping mechanism for PCMH evaluations is also common practice when attempting to differentiate outcomes by provider settings [59,60].

Our patient sample included all Medicaid beneficiaries that met the inclusion criteria for the HEDIS^®^ measure of follow-up care for attention-deficit/hyperactivity disorder (ADD) or medication management for people with asthma (MMA). The ADD measure is specific to recipients between 6 and 12 years of age who were newly prescribed ADHD medication. The MMA measure captures child recipients between the ages of 5 and 18. For this analysis, we collapsed the MMA age ranges into a single group. These measures were chosen because they represent conditions that result in frequent presentation in hospital emergency departments for disease-specific care that is often treatable in primary care settings [42,43]. Both measures were also chosen because they have a strong association with social vulnerability and residing in economically deprived areas and may be part of expanded risk factor adjustment metrics [55]. The study protocol (#Pro00093322) was reviewed and approved by the University’s Institutional Review Board (IRB) in accordance with 45 CFR 46.104(d)(4).

### PCMH attribution

SC Medicaid recipients select their primary care provider from five similarly designed Managed Care Organizations (MCO) or through fee-for-service (FFS) [61]. Over 80% of all Medicaid beneficiaries in SC are enrolled in an MCO, the majority of which include clinics that are NCQA-designated medical homes. This is in line national recommendations from the American Academy of Pediatrics (AAP), the National Association of Pediatric Nurse Practitioners (NAPNAP), and other organizations that all children have access to comprehensive, accessible, coordinated, and culturally appropriate care [62,63]. At the time of this study, there were no health (e.g., the number of chronic conditions) or plan-related (enrolled in MCO or FSS) restrictions on SC Medicaid beneficiaries for selecting a primary care provider whose practice was or was not a designated/accredited medical home.

It was not possible to identify from the claims data how each beneficiary initially elected to enroll with their primary care provider. However, it was possible to identify from the claims data whether the recipient’s health plan assigned them to a medical home as well as determine the designation status of the their primary care provider using their National Provider Identifier (NPI) and the NCAQ provider file. As such, we modeled two different depictions of PCMH attribution on pediatric ED visits: one based solely on attendance (i.e., attending a PCMH or not attending a PCMH) and one based on a 2x2 matrix of enrollment and attendance combinations (e.g., enrolled and attended a PCMH, enrolled and never attended a PCMH, etc.). We assessed both attribution types as the first method provides an account of care quality when based solely on patient attendance patterns that are typically available in health care registries, whereas the latter provides a finer depiction of a medical home effect in relation to geography. Beneficiaries that did not attend a PCMH or who were un-enrolled in a PCMH and never attended one for their primary care were used as the reference group.

### Emergency department visits

We used the New York University Emergency Department Algorithm (NYU-EDA) to define the level of acuity for all ED visits [64]. The NYU-EDA is a widely used measure of avoidable ED utilization [65–70]. The NYU-EDA classifies each ED visit probabilistically on a percentage basis into one of four categories: “non-emergent,” “emergent, but primary care treatable,” “emergent, but preventable/avoidable,” and “emergent, not preventable/avoidable”. All visits are classified based on the ICD diagnosis codes from the patient’s chart. The algorithm excludes uncommon diagnoses and treats mental health and substance abuse diagnoses separately from other causes. As it is possible to have probability scores assigned across all 4 categories, we used modifications to the event diagnosis groupings validated by Ballard et al (2010) to assign “non-emergent” (i.e., avoidable) and “emergent, but primary care treatable” (i.e., potentially avoidable) events into two distinct group, so long as the visit probability was greater than 0.50 for either event [71]. We excluded all visits that were the result of injury, or alcohol or drug problems since these conditions usually require ED services regardless of severity.

### Covariates

We were able to identify a limited set of covariate information from the claims data, including age, sex, race/ethnicity, co-morbidity classification, number of primary care visits, whether the ED visit occurred on a weekend, as well as whether the claim was attributed as fee-for-service (FFS). At the time of this study, the race/ethnicity field was not a mandatory data collection point by the South Carolina Medicaid agency. Due to small numbers and the large number of unknown/missing responses, we included only beneficiaries who reported their race/ethnicity as non-White Hispanic, Black, or Hispanic. Co-morbidities were defined using 3M^TM^ clinical risk group classification codes (CRG) [72]. CRGs are a population classification system derived from inpatient and ambulatory diagnosis and procedure codes, pharmaceutical data, as well as patient functional health status. CRG codes categorize patients into one of nine severity-adjusted groups, increasing in scale from heathy/non-users to catastrophic condition status. Through data linkages, we were able to obtain additional information pertaining to the patient’s neighborhood dwelling area type using modifications of the Rural Urban Commuting Area (RUCA) codes, the median household income of their residential census tract, as well as the proportion of medical homes relative to all primary care providers within their county of residence. RUCA codes were fixed using 2010 estimates whereas income and provider ratios were calculated yearly using American Community Survey and NCQA reports.

### Geographic assessment

To calculate geographic distances between beneficiaries and providers we built a composite geocoding algorithm that allowed for situs, linear, and area referencing of Medicaid claims data using a previously validated approach [73]. Prior to geocoding, we standardized each address file to US Postal Service mailing format to increase the likelihood of matching the provider address information to the street centerline file. Standardization was done using ZP4 address correction software. Next, we linked address information (e.g., street name, street suffix, ZIP code) from the claims database to street centerline data using ESRI’s Street Map Premium Address File. Average travel distance estimates (in miles) for all visits were then constructed for all unique pairings between the beneficiary’s residential address recorded in the claims database to the location where their primary care and ED visit occurred. The distance metric used in the regression model was the difference in distances between a beneficiary and their primary care provider versus the ED where the visit occurred. Negative values represented more convenient access to primary care providers, whereas positive values represented better proximity to hospitals. For ease of interpretation, relative differences in access were grouped using +/- 15 mile increments, with values of 0 representing no difference in distance between either location relative to the beneficiary’s residential address.

### Statistical analysis

In the unadjusted analysis, differences between means of continuous variables across PCMH attribution classifications were compared using ANOVA. Differences in proportions of categorical variables were examined using chi square tests. In the adjusted models, we measured the association between PCMH attribution and ED visits in two ways. First, we constructed a-spatial regression models to assess adjusted differences in event probabilities based on a patient’s PCMH attribution group type, with one model comparing ED visits based solely on PCMH attendance (e.g., attended a PCMH versus non-PCMH attendees) and another comparing visits based on the enrollment and attendance matrix (e.g., enrolled and attended a PCMH, enrolled and never attended a PCMH, etc.). Second, we expanded each model to account for the interaction with travel distance to providers. The spatial interaction models included all previously adjusted covariates.

We used a logistic regression analysis and marginal effects classifications to measure and compare the association between PCMH attribution type and the probability of an avoidable or potentially avoidable ED visit. Since beneficiaries may have had multiple ED visits during the study period as well as may not have been enrolled for the entire 3-year study period, we used a logistic model within a GEE to examine the probability of events while explicitly modeling the within-subject correlation. We initially selected a model using an independent correlation structure due to the small clusters and imbalanced design as well as the lack of complete confounder adjustment from the claims database. We ran quasi likelihood criterion tests to confirm this correlation structure was the best for model fit. All covariates that were statistically significant (p < 0.10) in bivariate analysis for each cohort were included in the regression models as a potential influence on ED visit risk. A full list of the covariates and regression coefficients from the models are provided in the online supplementary appendix. All statistical analyses were completed in STATA for Windows.

## Results

The final ADD patient sample consisted of 4,222 person-years from 2,959 unique non-Hispanic White, Black, and Hispanic Medicaid beneficiaries. From a total of 6,059 ED visits among the ADD cohort, 1,355 (22.3%) were classified as being non-emergent (i.e., avoidable) and 1,026 (16.9%) were classified as being emergent but primary care treatable (i.e., potentially avoidable), for an avoidable and potentially avoidable ED visit rate of 32.1 and 24.3 visits per 100 person-years, respectively. The final MMA patient sample consisted of 10,998 person-years from 6,390 unique non-Hispanic White, Black, and Hispanic Medicaid beneficiaries. There was a total of 20,462 ED visits, of which 4,205 (20.6%) and 3,515 (17.2%) were classified as avoidable or potentially avoidable, for a person-year rate of 38.2 and 31.9 avoidable and potentially avoidable ED visits per 100 person-years, respectively.

Tables 1 and 2 show that across all patients and PCMH attribution groups, the cohorts were dissimilar with respect to age, race/ethnicity, number of primary care provider visits, RUCA dwelling type, and county provider ratios (p < 0.05). The travel distances to primary care providers and hospitals were dissimilar among the MMA cohort for both PCMH attribution types, with medical home patients residing closer to all services irrespective of how enrollment and attendance groupings were defined (p < 0.01). There were no statistically significant differences in average travel differences to any service provider for the ADD cohort in the unadjusted comparisons.

**Table 1:** Person-years summary statistics for ADD outcomes.

	ADD cohort							
	PCMH attribution based on attendance			PCMH attribution based on enrollment and attendance				
Characteristic	PCMH (n = 1,140)	Non-PCMH (n = 1,519)	p	Group 1 (n = 1,320)	Group 22 (n = 924)	Group 3 (n = 199)	Group 4 (n = 516)	p value
Age (SD)	9.7 (1.7)	9.8 (1.8)	0.038	9.8 (1.8)	9.6 (1.7)	10.1 (1.7)	9.8 (1.7)	0.012
Sex (Male)	953 (66.2)	999 (65.8)	0.812	874 (66.2)	620 (67.1)	125 (62.8)	333 (64.5)	0.590
Race			0.000					0.000
non-Hispanic White	573 (39.8)	721 (47.5)		645 (48.9)	376 (40.7)	76 (38.2)	197 (38.2)	
Black	820 (56.9)	747 (49.2)		631 (47.8)	524 (56.7)	116 (58.3)	296 (57.4)	
Hispanic	47 (3.3)	51 (3.4)		44 (3.3)	24 (2.6)	7 (3.5)	23 (4.5)	
Clinical Risk Group			0.547					0.168
Healthy/non-users	310 (21.5)	362 (22.9)		304 (23.1)	207 (22.4)	58 (29.2)	103 (20.0)	
History of significant acute disease	50 (3.5)	56 (3.7)		54 (4.1)	29 (3.1)	2 (1.0)	21 (4.1)	
Single minor chronic disease	594 (41.3)	627 (41.4)		546 (41.5)	394 (42.7)	81 (40.7)	200 (38.8)	
Minor chronic disease in multiple organ systems	53 (3.7)	46 (3.0)		41 (3.1)	30 (3.3)	5 (2.5)	23 (4.5)	
Single dominant or moderate chronic disease	319 (22.2)	326 (21.5)		282 (21.4)	188 (20.4)	44 (22.1)	131 (25.4)	
Significant chronic disease in multiple organ systems	109 (7.6)	97 (6.4)		88 (21.4)	72 (7.8)	9 (4.5)	37 (7.2)	
Dominant chronic disease in 3+ organ systems	1 (0.1)	0 (0.0)		0 (0.0)	1 (0.1)	0 (0.0)	0 (0.0)	
Dominant and metastatic malignancies	1 (0.1)	0 (0.0)		0 (0.0)	0 (0.0)	0 (0.0)	1 (0.2)	
Catastrophic condition status	2 (0.1)	1 (0.1)		1 (0.1)	2 (0.2)	0 (0.0)	0 (0.0)	
FFS plan	105 (7.3)	142 (9.4)	0.043	139 (10.5)	98 (10.6)	3 (1.5)	7 (1.4)	0.000
Primary care visits (SD)	4.2 (2.3)	3.7 (2.0)	0.000	3.8 (2.1)	4.2 (2.3)	3.5 (1.9)	4.2 (2.1)	0.000
Weekend ED visits	403 (28.0)	410 (27.0)	0.545	351 (26.6)	258 (27.9)	59 (29.7)	145 (28.1)	0.757
Dwelling location			0.000					0.000
Urban	643 (44.9)	504 (33.3)		423 (32.1)	398 (43.4)	81 (40.7)	245 (47.8)	
Suburban	321 (22.4)	509 (33.6)		448 (34.0)	200 (21.8)	61 (30.7)	121 (23.6)	
Rural	467 (32.6)	503 (33.2)		446 (33.9)	320 (34.9)	57 (28.6)	147 (28.7)	
Median household income of census tract (SD)	40,563 (13,960)	40,595 (14,376)	0.928	40,725 (14,653)	40,077 (12,829)	39,772 (12,454)	41,439 (15,765)	0.050
County PCMH proportion	21.5 (16.6)	16.3 (15.3)	0.000	15.5 (15.3)	20.8 (16.3)	21.8 (14.3)	22.7 (16.8)	0.000
Mean distance to PCP (SD)	42.7 (54.6)	43.5 (53.9)	0.535	43.7 (57.1)	41.7 (48.8)	42.6 (23.9)	44.3 (63.4)	0.565
Mean distance to ED (SD)	18.6 (55.1)	18.5 (47.0)	0.955	18.5 (49.5)	17.9 (50.7)	18.2 (26.7)	19.8 (62.3)	0.803
ED visits								
Non-emergent (avoidable)	616 (21.0)	739 (23.6)	0.014	621 (23.0)	383 (20.4)	118 (28.0)	233 (22.1)	0.006
Emergent/primary care treatable (potentially avoidable)	514 (17.5)	512 (16.4)	0.235	442 (16.4)	320 (17.0)	70 (16.6)	194 (18.4)	0.512

PCMH attribution groups: (1) un-enrolled and never attended; (2) un-enrolled and attended, (3) enrolled and never attended, (4) enrolled and always attended; PCP = primary care provider; ED = emergency department; SD = standard deviation (from mean); FFS = fee-for-service.

**Table 2:** Person-years summary statistics for MMA outcomes.

	ADD cohort							
	PCMH attribution based on attendance			PCMH attribution based on enrollment and attendance				
Characteristic	PCMH (n = 3,274)	Non-PCMH (n = 3,116)	p	Group 1 (n = 2,625)	Group 22 (n = 1,762)	Group 3 (n = 491)	Group 4 (n = 1,512)	p value
Age (SD)	9.8 (3.5)	10.2 (3.6)	0.000	10.2 (3.6)	9.7 (3.5)	10.3 (3.6)	9.8 (3.5)	0.000
Sex (Male)	1,902 (58.1)	1,800 (57.8)	0.791	1,518 (57.8)	1,011 (57.4)	282 (57.4)	891 (58.9)	0.826
Race			0.098					0.000
non-Hispanic White	1,095 (33.5)	1,101 (35.3)		953 (36.3)	593 (33.7)	148 (30.1)	502 (33.2)	
Black	1,952 (59.6)	1,832 (58.8)		1,529 (58.3)	1,070 (60.7)	303 (61.7)	882 (58.3)	
Hispanic	227 (6.9)	183 (5.9)		143 (5.5)	99 (5.6)	40 (8.2)	128 (8.5)	
Clinical Risk Group			0.019					0.036
Healthy/non-users	267 (8.2)	291 (9.3)		243 (9.3)	128 (7.3)	48 (9.8)	139 (9.2)	
History of significant acute disease	277 (8.5)	327 (10.5)		269 (10.3)	140 (8.0)	58 (11.8)	137 (9.1)	
Single minor chronic disease	155 (4.7)	168 (5.4)		138 (5.3)	86 (4.9)	30 (6.1)	69 (4.6)	
Minor chronic disease in multiple organ systems	14 (0.4)	15 (0.5)		12 (0.5)	10 (0.6)	3 (0.6)	4 (0.3)	
Single dominant or moderate chronic disease	1,970 (60.2)	1,818 (58.3)		1,526 (58.1)	1,072 (60.8)	292 (59.5)	898 (59.4)	
Significant chronic disease in multiple organ systems	585 (17.9)	492 (15.8)		433 (16.5)	322 (18.3)	59 (12.0)	263 (17.4)	
Dominant chronic disease in 3+ organ systems	2 (0.1)	0 (0.0)		0 (0.0)	2 (0.1)	0 (0.0)	0 (0.0)	
Dominant and metastatic malignancies	1 (0.0)	2 (0.1)		2 (0.1)	0 (0.0)	0 (0.0)	1 (0.1)	
Catastrophic condition status	3 (0.1)	3 (0.1)		2 (0.1)	2 (0.1)	1 (0.2)	1 (0.1)	
FFS plan	172 (5.3)	178 (5.7)	0.420	170 (6.5)	146 (8.3)	8 (1.6)	26 (1.7)	0.000
Primary care visits (SD)	5.7 (3.5)	4.6 (2.5)	0.000	4.6 (2.4)	5.6 (3.5)	4.5 (2.6)	5.8 (3.6)	0.000
Weekend ED visits	1,061 (32.1)	917 (29.4)	0.010	762 (29.0)	586 (33.3)	155 (31.6)	475 (31.4)	0.027
Dwelling location			0.000					0.000
Urban	1,762 (53.9)	1,330 (42.7)		1,065 (40.6)	921 (52.3)	265 (54.0)	841 (55.6)	
Suburban	711 (21.7)	897 (28.8)		787 (30.0)	379 (21.5)	110 (22.4)	332 (22.0)	
Rural	799 (24.4)	889 (28.5)		773 (30.5)	460 (26.1)	116 (23.6)	339 (22.4)	
Median household income of census tract (SD)	42,281 (14,801)	40,508 (14,107)	0.000	40,422 (13,908)	41,988 (14,955)	40,968 (15,125)	42,636 (14,607)	0.000
County PCMH proportion	21.1 (16.0)	16.0 (15.0)	0.000	15.3 (14.8)	21.2 (15.9)	19.4 (16.0)	21.1 (16.0)	0.000
Mean distance to PCP (SD)	36.8 (26.7)	41.6 (31.4)	0.000	41.3 (29.8)	37.6 (28.5)	43.1 (39.2)	35.8 (24.1)	0.000
Mean distance to ED (SD)	15.8 (28.3)	17.3 (40.6)	0.003	16.8 (28.7)	16.2 (22.9)	19.4 (78.0)	15.2 (33.6)	0.000
ED visits								
Non-emergent (avoidable)	2,121 (20.8)	2,084 (20.3)	0.359	1,713 (19.8)	1,161 (20.8)	371 (22.9)	960 (20.8)	0.028
Emergent/primary care treatable (potentially avoidable)	1,730 (17.0)	1,785 (17.4)	0.441	1,508 (17.4)	947 (17.0)	277 (17.1)	783 (17.0)	0.877

PCMH attribution groups: (1) un-enrolled and never attended; (2) un-enrolled and attended, (3) enrolled and never attended, (4) enrolled and always attended; PCP = primary care provider; ED = emergency department; SD = standard deviation (from mean); FFS = fee-for-service.

A-spatial adjusted predicted probabilities for ED visit type by clinic attribution grouping for each cohort are shown in Table 3. Among the ADD cohort, the adjusted predicted probability of experiencing an avoidable or potentially avoidable visit by clinic attribution grouping ranged from 0.205 to 0.282 and from 0.162 to 0.174, respectively. The 2.4 percentage point reduction in risk of avoidable ED visits among children in the ADD cohort who attended a PCMH versus those who did not was statistically significant (p < 0.01). The 1.1 percentage point increase in risk of a potentially avoidable ED visit among children in the ADD cohort who attended a PCMH was not statistically significant. When PCMH attribution was based on enrollment and attendance, children in the ADD cohort who were enrolled in a PCMH but never attended one for their primary care exhibited a 5.4 percentage point increase in an avoidable ED visit (p < 0.05) compared to children who were un-enrolled in a medical home and never attended one for primary care (reference group). The 2.4 percentage point decrease in risk for avoidable visits among children that were enrolled and attended a medical home was not statistically significant from the reference group. Among the MMA cohort, the adjusted probability for avoidable or potentially avoidable visits ranged from 0.196 to 0.226 and from 0.167 to 0.173 across all attribution groups, respectively. Children in the MMA cohort who were enrolled in a PCMH and never attended one for primary care exhibited a 3.0 percentage point increase in risk of an avoidable ED visit compared to children in the reference group (p < 0.05). No other comparisons were statistically significant. Regression coefficients for all models are provided in the supplementary online appendix.

**Table 3:** A-spatial comparisons of predicted probabilities in ED visit category by medical home attribution grouping type.

	ADD Cohort		MMA Cohort	
	Avoidable Visits	Potentially Avoidable Visits	Avoidable Visits	Potentially Avoidable Visits
**PCMH grouping type 1**				
Non-PCMH	0.235 (0.008)**	0.163 (0.007)**	0.201 (0.004)**	0.172 (0.004)**
PCMH	0.211 (0.008)**	0.174 (0.007)**	0.205 (0.004)**	0.168 (0.004)**
**Δ Differences in margins (type 1)**				
PCMH versus non-PCMH	-0.024 (0.011)*	0.011 (0.010)	0.004 (0.006)	-0.004 (0.006)
**PCMH grouping type 2**				
Un-enrolled + never attended (1)	0.229 (0.009)**	0.163 (0.008)**	0.196 (0.005)**	0.173 (0.005)**
Un-enrolled + always attended (2)	0.205 (0.010)**	0.169 (0.009)**	0.206 (0.006)**	0.167 (0.005)**
Enrolled + never attended (3)	0.282 (.022)**	0.163 (0.019)**	0.226 (0.016)**	0.169 (0.010)**
Enrolled + always attended (4)	0.223 (0.013)**	0.183 (0.012)**	0.204 (0.007)**	0.169 (0.006)**
**Δ Differences in margins (type 2)**				
group 2 versus group 1	-0.006 (0.016)	0.020 (0.014)	0.007 (0.008)	-0.004 (0.008)
group 3 versus group 1	0.054 (0.024)*	0.004 (0.020)	0.030 (0.012)*	-0.004 (0.011)
group 4 versus group 1	-0.024 (0.013)	0.006 (0.012)	0.009 (0.008)	-0.006 (0.007)

PCMH attribution groups: (1) un-enrolled and never attended; (2) un-enrolled and attended, (3) enrolled and never attended, (4) enrolled and always attended. All group comparisons in bold are statistically significant at

** p < 0.01,

* p < 0.05.

Table 4 shows the results of models that included the spatial interactions. After accounting for relative differences in proximity to providers, the 2.4 percentage point decrease in risk of avoidable ED visits among children in the ADD cohort shown in Table 3 increased to 3.9 to 7.2 percentage points as the relative proximity to primary care providers over hospitals improved (p < 0.05). The differences in risk remained 3.1 percentage points lower so long as the distance to the medical home was not 15 miles further to access than the hospital (p < 0.01). In contrast to the a-spatial model that found no difference in risk of potentially avoidable visits by PCMH attribution type, children in the ADD cohort that attended medical homes exhibited a 3.2 to 7.6 percentage point increase in risk of a potentially avoidable visit so long as proximity to primary care providers was more convenient than EDs (p < 0.05). There were no differences in risk of an avoidable ED visit among the ADD cohort when proximity to hospitals was the most convenient.

**Table 4:** Comparisons of predicted probabilities in ED visits for ADD outcomes by medical home attribution type after accounting for geographic proximity to providers.

	ADD Cohort													
	Avoidable Visits							Potentially Avoidable Visits						
	(Greater proximity to PCP)			Equal distance	(Greater proximity to ED)			(Greater proximity to PCP)			Equal distance	(Greater proximity to ED)		
	60 miles	30 miles	15 miles	0 miles	15 miles	30 miles	60 miles	60 miles	30 miles	15 miles	0 miles	15 miles	30 miles	60 miles
**PCMH attribution (type 1)**														
Non-PCMH	0.255 (0.025)	0.248 (0.017)	0.245 (0.014)	0.242 (0.011)	0.239 (0.009)	0.236 (0.008)	0.230 (0.012)	0.127 (0.020)	0.139 (0.015)	0.145 (0.012)	0.152 (0.010)	0.158 (0.008)	0.165 (0.007)	0.180 (0.013)
PCMH	0.182 (0.023)	0.192 (0.016)	0.197 (0.013)	0.203 (0.010)	0.208 (0.008)	0.213 (0.008)	0.225 (0.014)	0.204 (0.025)	0.194 (0.012)	0.189 (0.013)	0.184 (0.010)	0.179 (0.008)	0.174 (0.008)	0.165 (0.012)
**Δ Differences in margins**	-0.072 (0.034)*	-0.056 (0.024)*	-0.048 (0.019)*	-0.039 (0.015)**	-0.031 (0.012)**	-0.023 (0.012)	-0.005 (0.018)	0.076 (0.032)*	0.055 (0.022)*	0.043 (0.018)*	0.032 (0.014)*	0.021 (0.011)	0.009 (0.011)	-0.014 (0.017)
**PCMH attribution (type 2)**														
Unenrolled + never went (1)	0.253 (0.027)	0.245 (0.019)	0.241 (0.015)	0.237 (0.011)	0.232 (0.009)	0.228 (0.009)	0.221 (0.013)	0.134 (0.023)	0.144 (0.017)	0.149 (0.013)	0.154 (0.010)	0.159 (0.008)	0.165 (0.008)	0.176 (0.013)
Unenrolled + always went (2)	0.173 (0.026)	0.184 (0.019)	0.190 (0.016)	0.195 (0.012)	0.201 (0.010)	0.207 (0.010)	0.219 (0.016)	0.193 (0.029)	0.186 (0.020)	0.182 (0.015)	0.178 (0.012)	0.174 (0.010)	0.171 (0.010)	0.165 (0.015)
Enrolled + never went (3)	0.259 (0.067)	0.268 (0.047)	0.272 (0.037)	0.277 (0.029)	0.282 (0.023)	0.286 (0.022)	0.296 (0.037)	0.082 (0.041)	0.105 (0.034)	0.119 (0.030)	0.134 (0.024)	0.151 (0.020)	0.170 (0.020)	0.212 (0.041)
Enrolled + always went (4)	0.199 (0.045)	0.208 (0.032)	0.212 (0.025)	0.216 (0.019)	0.220 (0.014)	0.225 (0.014)	0.233 (0.025)	0.227 (0.047)	0.210 (0.030)	0.202 (0.023)	0.195 (0.016)	0.187 (0.012)	0.180 (0.012)	0.166 (0.019)
**Δ Differences in margins**														
group 2 versus group 1	-0.079 (0.038)*	-0.061 (0.027)*	-0.051 (0.021)*	-0.041 (0.017)*	-0.031 (0.014)*	-0.021 (0.013)	-0.002 (0.021)	0.059 (0.037)	0.042 (0.026)	0.033 (0.020)	0.025 (0.016)	0.016 (0.013)	0.007 (0.013)	-0.011 (0.020)
group 3 versus group 1	0.006 (0.072)	0.023 (0.050)	0.032 (0.040)	0.041 (0.031)	0.049 (0.025)*	0.058 (0.024)*	0.075 (0.039)	-0.052 (0.046)	-0.039 (0.038)	-0.030 (0.033)	-0.020 (0.026)	-0.008 (0.021)	0.005 (0.021)	0.036 (0.044)
group 4 versus group 1	-0.054 (0.053)	-0.037 (0.037)	-0.029 (0.028)	-0.020 (0.022)	-0.012 (0.017)	-0.004 (0.016)	0.013 (0.028)	0.093 (0.052)	0.067 (0.034)*	0.054 (0.026)*	0.041 (0.019)*	0.028 (0.015)	0.015 (0.014)	-0.010 (0.023)

PCMH attribution groups: (1) un-enrolled and never attended; (2) un-enrolled and attended, (3) enrolled and never attended, (4) enrolled and always attended. All group comparisons in bold are statistically significant at

** p < 0.01,

* p < 0.05.

When assessed by PCMH attribution and enrollment type, children in the ADD cohort who were enrolled in a medical home, but never attended one for their primary care services exhibited a 4.9 to 5.8 percentage point increase in risk of an avoidable ED visit relative to children in the reference group so long as proximity to primary care providers was more convenient (p < 0.05), whereas children who attended medical homes even though they were un-enrolled in one through their MCO showed a 7.9 to 3.1 percentage point decrease in risk of avoidable ED visits relative to children in the reference group (p < 0.05). Similar trends in risk were found among children who were enrolled and attended medical homes, but the reductions in risk were not statistically significant.

As shown in Table 5, there were few statistically significant associations between PCMH attribution type and risk of avoidable and potentially avoidable ED visits among the MMA cohort after including the spatial interactions. As in the a-spatial comparisons, persons who were enrolled in a medical home, but did not attend one for their primary care exhibited an increased risk of avoidable ED visits, ranging in magnitude from 2.9 to 3.7 percentage points, but only when the proximity to the hospital was closer than their primary care provider (p < 0.05). No other spatial interactions were statistically significant.

**Table 5:** Comparisons of predicted probabilities in ED visits for MMA outcomes by medical home attribution type after accounting for geographic proximity to providers.

	MMA Cohort													
	Avoidable Visits							Potentially Avoidable Visits						
	(Greater proximity to PCP)			Equal distance	(Greater proximity to ED)			(Greater proximity to PCP)			Equal distance	(Greater proximity to ED)		
	60 miles	30 miles	15 miles	0 miles	15 miles	30 miles	60 miles	60 miles	30 miles	15 miles	0 miles	15 miles	30 miles	60 miles
**PCMH attribution (type 1)**														
Non-PCMH	0.191 (0.010)	0.195 (0.007)	0.197 (0.006)	0.199 (0.005)	0.201 (0.005)	0.203 (0.004)	0.207 (0.006)	0.171 (0.011)	0.173 (0.007)	0.174 (0.006)	0.174 (0.005)	0.175 (0.004)	0.176 (0.004)	0.177 (0.006)
PCMH	0.209 (0.012)	0.209 (0.008)	0.208 (0.007)	0.208 (0.005)	0.208 (0.005)	0.208 (0.005)	0.208 (0.007)	0.174 (0.009)	0.173 (0.006)	0.172 (0.005)	0.171 (0.005)	0.171 (0.004)	0.170 (0.004)	0.168 (0.006)
**Δ Differences in margins**	0.018 (0.015)	0.014 (0.011)	0.012 (0.009)	0.010 (0.007)	0.007 (0.006)	0.005 (0.007)	0.001 (0.009)	0.003 (0.014)	0.000 (0.010)	-0.001 (0.008)	-0.002 (0.007)	-0.005 (0.006)	-0.006 (0.006)	-0.009 (0.008)
**PCMH attribution (type 2)**														
Unenrolled + never went (1)	0.190 (0.010)	0.192 (0.008)	0.194 (0.006)	0.195 (0.005)	0.196 (0.005)	0.198 (0.005)	0.201 (0.006)	0.167 (0.010)	0.170 (0.008)	0.172 (0.006)	0.173 (0.005)	0.175 (0.005)	0.177 (0.005)	0.180 (0.007)
Unenrolled + always went (2)	0.198 (0.017)	0.202 (0.012)	0.204 (0.009)	0.206 (0.007)	0.208 (0.006)	0.210 (0.006)	0.214 (0.010)	0.164 (0.014)	0.166 (0.010)	0.167 (0.008)	0.168 (0.006)	0.169 (0.006)	0.170 (0.006)	0.172 (0.008)
Enrolled + never went (3)	0.207 (0.014)	0.214 (0.018)	0.218 (0.016)	0.222 (0.014)	0.226 (0.012)	0.230 (0.012)	0.238 (0.015)	0.186 (0.032)	0.181 (0.022)	0.179 (0.017)	0.177 (0.014)	0.175 (0.011)	0.173 (0.010)	0.168 (0.015)
Enrolled + always went (4)	0.217 (0.014)	0.213 (0.010)	0.212 (0.009)	0.210 (0.007)	0.209 (0.007)	0.207 (0.007)	0.204 (0.010)	0.181 (0.010)	0.177 (0.008)	0.176 (0.007)	0.174 (0.006)	0.172 (0.006)	0.170 (0.006)	0.166 (0.007)
**Δ Differences in margins**														
group 2 versus group 1	0.008 (0.020)	0.009 (0.014)	0.010 (0.011)	0.011 (0.009)	0.011 (0.008)	0.012 (0.008)	0.013 (0.012)	-0.003 (0.017)	-0.004 (0.012)	-0.005 (0.010)	-0.005 (0.008)	-0.006 (0.007)	-0.007 (0.007)	-0.008 (0.011)
group 3 versus group 1	0.017 (0.026)	0.022 (0.020)	0.025 (0.017)	0.027 (0.015)	0.029 (0.013)*	0.032 (0.013)*	0.037 (0.016)*	0.018 (0.033)	0.011 (0.023)	0.007 (0.018)	0.004 (0.015)	-0.000 (0.012)	-0.004 (0.011)	-0.012 (0.016)
group 4 versus group 1	0.027 (0.017)	0.021 (0.013)	0.018 (0.010)	0.015 (0.009)	0.012 (0.009)	0.009 (0.008)	0.003 (0.001)	0.015 (0.014)	0.008 (0.011)	0.004 (0.009)	0.000 (0.008)	-0.003 (0.008)	-0.007 (0.008)	-0.014 (0.010)

PCMH attribution groups: (1) un-enrolled and never attended; (2) un-enrolled and attended, (3) enrolled and never attended, (4) enrolled and always attended. All group comparisons in bold are statistically significant at

** p < 0.01,

* p < 0.05.

Prior to the analysis, we conducted a sensitivity analysis of ED visits by primary diagnosis codes and PCMH attribution group. For the ADD cohort, the top 10 primary diagnosis codes were identical for 90% of all visits, with the leading causes attributed to J02.9 (Acute pharyngitis, unspecified); J06.9(Acute upper respiratory infection, unspecified); and J02.0 (Streptococcal pharyngitis). Depending on the group, the top visit causes contributed to 20% to 25% of all visits. The diagnosis codes were identical for 88% of the leading visit causes for the MMA cohort, with most claims attributed to J45.901 (Unspecified asthma with (acute) exacerbation); J06.9 (Acute upper respiratory infection, unspecified); and J45.909 (Unspecified asthma, uncomplicated). Depending on group, the top visit causes accounted for 32% - 34% of all visits, respectively.

## Discussion

This study analyzed a subset of South Carolina Medicaid pediatric claims data for beneficiaries having a pre-existing diagnosis of asthma or attention-deficit/hyperactivity disorder and assessed different a-spatial and spatial interactions of visit risk based on whether or not the beneficiary attended a designated medical home. We assessed two different depictions of a beneficiary’s PCMH attribution type: one based completely on attendance, and one based on enrollment and attendance records. We found that geographical location between patients, primary care providers, and hospitals does play a role in ED utilization for some pediatric groups with pre-existing chronic illnesses. We also found that these associations varied by PCMH attribution type. Many of the interactions showed that a-spatial findings were driven by instances where children had better access to medical homes versus hospitals. The intersection of these findings suggests that for some pediatric patient populations with pre-existing illnesses, a medical home effect on ED utilization rates may be contingent on where primary providers are located relative to hospitals. These findings provide some evidence that previous pediatric assessments of ED utilizations may be under-counting the benefit of medical home-modeled care owing to a distance decay effect patient’s and providers. That medical home effectiveness gets diluted owing to geographic proximity to providers may be particularly relevant for future evaluations owing to recent findings that primary care practices that elect to transition into designated medical homes tend to locate in more socioeconomically advantaged communities [74].

However, despite these findings, the effects of geographic location on risk reductions attributable to medical home attendance were not consistently found for both cohorts. Contextually, the mixed findings raise questions as to why PCMH attendance would exhibit a benefit to some ED visit types and opposite trends for other visit types once proximity to providers was assessed. That the spatial interactions either increased the a-spatial finding that PCMH attendance lowered risk of ED utilization or revealed trends that were not previously observed suggests that the significance of geographic location is more meaningful than spurious. One explanation might stem from the differences in proximity to providers within the MMA cohort, as its medical home attendees had to travel approximately 5 to 10 miles less to access PCMHs compared to children in the ADD cohort. Patient morbidity, provider recommendations, or time of day may have also accounted for differences in utilization trends for emergent but potentially avoidable visits. Whether these patterns are repeated in other patient cohorts or among adult ED utilization patterns is an important topic for future research.

Of the recent summaries and meta-analyses of medical home evaluations, the reviews conducted by Sinaiko *et al* (2017), Friedberg *et al* (2014), and Jackson *et al* (2013) have shown variation in overall improvements in primary care utilization, ED visits, as well as inpatient hospital admissions [41,75,76]. Factors such as practice size, patient mix, and practice ownership contribute to observed heterogeneity in study findings. Although evidence exists that geography escalates variation in healthcare access and outcomes [77–83], geographic location as a potential cause for variation in PCMH success has not been rigorously examined. This gap is significant as needs-based medical assistance programs such as the Centers for Medicare and Medicaid Services (CMS) rely on information technology platforms such as GIS to evaluate whether Medicaid beneficiaries have timely and adequate access to the providers and services that participate in its network [58,84]. Our findings that higher utilization patterns may have been driven by relative distances to providers or that non-emergent visits were consistently higher among patients that were documented as enrolled in a PCMH but never attended one for their primary care may point to specific instances where network adequacy thresholds that account for relative distances between providers might be warranted. Neither of these findings could have been uncovered without including spatial interactions in the models.

The HEDIS measures chosen for analysis were selected based on the relationship between comorbid conditions and hospital emergency department encounters that are often avoidable or treatable in primary care settings [42,43]. Its use also helped establish baseline similarities across the patient panels that made it possible to exploit differences in outcomes attributed to medical home status and/or geography. Although our study was limited in the number of quality indicators available from the encounter data, our findings are encouraging from the standpoint that additional benefits (or lack thereof) of medical home attribution can be identified for patient populations once details of health services locations are included in the model. Future work could build on this approach to assess whether the geographic location of medical homes modifies other performance measures.

The observations from this study have some noteworthy limitations. Firstly, access to health care services is a multidimensional concept and influenced by factors that have both spatial and a-spatial dimensions. Although our evaluation did allow us to control for key factors required for meaningful risk-adjustment, other clinical and patient-reported measures that we were unable to obtain may further delineate the benefits attributed to medical home access. For example, we could not control for factors such as wait times, historical service use, time or day, or patient satisfaction with ED care versus primary care in our analysis, all of which may have helped to further contextualize differences in use. Similarly, although many state Medicaid agencies are experimenting with some version of PCMH transformation, the varied nature of state Medicaid characteristics make national geographic comparisons difficult to construct and we cannot say whether these trends over or underestimate its effect. Additional state-based tests would be beneficial for context validity as well as for defining appropriate access thresholds for other performance measures. With respect to our spatial approach, the primary limitation of assessing relative differences to providers is that we did not focus on exact distances to care. For example, in our model someone could have been assigned into the 0–15 mile grouping if they lived 20 miles from the ED or 200 miles from the ED, so long as the difference in proximity to their primary care provider remained within 15 miles. Similarly, our analysis may have been biased owing to the lack of information on PCMH enrollment and attendance in the claims data, although we were able to circumvent some of these issues using the enrollment and attendance matrix. Similarly, we could not confirm whether all beneficiaries initiated their care from their place of residence or how they traveled to obtain care (e.g., car, bus, walk), but we could confirm that all claims represented physical encounters at primary care clinics and hospitals. Another limitation is that our analysis may have muted disparities that might have emerged had we included privately insured populations. However, we did find that differences in outcomes among some of the most socially vulnerable populations in the state could be demarcated once the models accounted for relative distances populations must travel to obtain health care services.

## Conclusion

In the age of patient-centered medicine, GIS technology is becoming even more important for optimizing case management (e.g., list of available transportation resources) as well as enhancing patient experience with care (e.g., providing web-based service locations of nearby providers). Although geographic variation is an accepted phenomenon in health care services research, the findings in this study illustrate that health care performance measures can be enhanced by knowing how far patients must travel in order to obtain services. Although medical home attendance may not be consistently associated with lower ED visits for all patient groups, our findings suggest that the lack of spatial information may be one factor why the anticipated effects of some PCMH innovations is often muted.

## Supporting information

S1 Table Adjusted GEE coefficients for ED visits where PCMH attribution was based on attendance data. Covariates were included in the models if bivariate comparisons had p-values at or below 0.10.
(DOCX)

S2 Table Adjusted GEE coefficients for ED visits where PCMH attribution was based on attendance and enrollment data. Covariates were included in the models if bivariate comparisons had p-values at or below 0.10.
(DOCX)

## Abbreviations

ADD HEDIS: measure for Follow-Up Care for Children Prescribed ADHD Medication
MMA HEDIS: measure Medication Management for People with Asthma
FFS A: Medicaid beneficiary who is enrolled under a fee for service payment plan
NCQA: National Committee for Quality Assurance
GIS: geographic information systems/science
HEDIS: Healthcare Effectiveness Data and Information Set
PCMH: Patient-Centered Medical Homes
Non-PCMH: Medicaid beneficiaries who were un-enrolled and did not attend a designated medical home

## References

[1] Institute of Medicine, Unequal Treatment: Confronting Racial and Ethnic Disparities in Health Care, ed. Smedley B.D., Stith A.Y., and Nelson A.R.. 2003, Washington (DC): National Academies Press. 25032386

[2] Admon L.K., et al., Insurance Coverage and Perinatal Health Care Use Among Low-Income Women in the US, 2015–2017. JAMA Netw Open, 2021. 4(1): p. e2034549. doi: 10.1001/jamanetworkopen.2020.34549 33502480 PMC7841453

[3] Dess R.T., et al., Association of Black Race With Prostate Cancer–Specific and Other-Cause Mortality. JAMA Oncology, 2019. 5(7): p. 975–983. doi: 10.1001/jamaoncol.2019.0826 31120534 PMC6547116

[4] Buchmueller T.C. and Levy H.G., The ACA’s Impact On Racial And Ethnic Disparities In Health Insurance Coverage And Access To Care. Health Affairs, 2020. 39(3): p. 395–402. doi: 10.1377/hlthaff.2019.01394 32119625

[5] VanderWielen L.M., et al., Not Near Enough: Racial and Ethnic Disparities in Access to Nearby Behavioral Health Care and Primary Care. Journal of Health Care for the Poor and Underserved, 2015. 26(3): p. 1032–1047. doi: 10.1353/hpu.2015.0083 26320931 PMC4962556

[6] Sun R., Karaca Z., and Wong H.S., Characteristics of Homeless Individuals Using Emergency Department Services in 2014: Statistical Brief #229, in Healthcare Cost and Utilization Project (HCUP) Statistical Briefs. 2006: Rockville (MD). 29400922

[7] Seligman H.K., et al., Exhaustion of food budgets at month’s end and hospital admissions for hypoglycemia. Health Aff (Millwood), 2014. 33(1): p. 116–23. doi: 10.1377/hlthaff.2013.0096 24395943 PMC4215698

[8] Hakulinen C., et al., Social isolation and loneliness as risk factors for myocardial infarction, stroke and mortality: UK Biobank cohort study of 479 054 men and women. Heart, 2018. 104(18): p. 1536–1542. doi: 10.1136/heartjnl-2017-312663 29588329

[9] Bell N., et al., Does direct transport to provincial burn centres improve outcomes? A spatial epidemiology of severe burn injury in British Columbia, 2001–2006. Canadian Journal of Surgery, 2012. 55(2): p. 110–116. doi: 10.1503/cjs.014708 22564514 PMC3310766

[10] Kelly C., et al., Are differences in travel time or distance to healthcare for adults in global north countries associated with an impact on health outcomes? A systematic review. BMJ Open, 2016. 6(11): p. e013059. doi: 10.1136/bmjopen-2016-013059 27884848 PMC5178808

[11] Arcury T.A., et al., The effects of geography and spatial behavior on health care utilization among the residents of a rural region. Health Services Research, 2005. 40(1): p. 135–155. doi: 10.1111/j.1475-6773.2005.00346.x 15663706 PMC1361130

[12] Chen B.K., et al., Travel distance and sociodemographic correlates of potentially avoidable emergency department visits in California, 2006–2010: an observational study. International journal for equity in health, 2015. 14: p. 30–30. doi: 10.1186/s12939-015-0158-y 25889646 PMC4391132

[13] Durand A.C., et al., Nonurgent patients in emergency departments: rational or irresponsible consumers? Perceptions of professionals and patients. BMC Res Notes, 2012. 5: p. 525. doi: 10.1186/1756-0500-5-525 23006316 PMC3515357

[14] Hoot N.R. and Aronsky D., Systematic review of emergency department crowding: causes, effects, and solutions. Ann Emerg Med, 2008. 52(2): p. 126–36. doi: 10.1016/j.annemergmed.2008.03.014 18433933 PMC7340358

[15] Owens P.L., et al., Annual report on health care for children and youth in the United States: focus on injury-related emergency department utilization and expenditures. Ambulatory pediatrics: the official journal of the Ambulatory Pediatric Association, 2008. 8(4): p. 219–240.e17. doi: 10.1016/j.ambp.2008.03.032 18644545

[16] Dowd B., et al., Emergency department utilization as a measure of physician performance. Am J Med Qual, 2014. 29(2): p. 135–43. doi: 10.1177/1062860613487196 23687240

[17] Beydon N., et al., Avoidable Emergency Visits for Acute Asthma in Children: Prevalence and Risk Factors. Pediatric Allergy Immunology and Pulmonology, 2016. 29(3): p. 130–136. doi: 10.1089/ped.2016.0646 35923055

[18] Akinbarni, L., et al., Health Care Use, and Mortality in the United States, 2001–2010, in National Center for Health Statistics Data Brief 94. 2012: Available online at: https://www.cdc.gov/nchs/data/databriefs/db94.pdf.

[19] Lynch S., et al., Toward Effective Utilization of the Pediatric Emergency Department: The Case of ADHD. Soc Work Public Health, 2016. 31(1): p. 9–18. doi: 10.1080/19371918.2015.1087909 26667350

[20] Cydulka R.K., et al., Inadequate follow-up controller medications among patients with asthma who visit the emergency department. Ann Emerg Med, 2005. 46(4): p. 316–22. doi: 10.1016/j.annemergmed.2004.12.024 16187464

[21] Soto E.C., et al., Frequency and correlates of inappropriate pediatric psychiatric emergency room visits. J Clin Psychiatry, 2009. 70(8): p. 1164–77. doi: 10.4088/JCP.08m04839 19758526

[22] AHRQ. Statistical Brief #227. Healthcare Cost and Utilization Project (HCUP) 2017 [cited 2021 January 15]; Available from: www.hcup-us.ahrq.gov/reports/statbriefs/sb227-Emergency-Department-Visit-Trends.jsp.

[23] Phillips R.L., et al., Cost, Utilization, and Quality of Care: An Evaluation of Illinois’ Medicaid Primary Care Case Management Program. Annals of Family Medicine, 2014. 12(5): p. 408–417. doi: 10.1370/afm.1690 25354404 PMC4157977

[24] Timmins L., Peikes D., and McCall N., Pathways to reduced emergency department and urgent care center use: Lessons from the comprehensive primary care initiative. Health Serv Res, 2020. 55(6): p. 1003–1012. doi: 10.1111/1475-6773.13579 33258126 PMC7704466

[25] Rosser W.W., et al., Progress of Ontario’s Family Health Team model: a patient-centered medical home. Ann Fam Med, 2011. 9(2): p. 165–71. doi: 10.1370/afm.1228 21403144 PMC3056865

[26] Chimento G.F. and Thomas L.C., The Perioperative Surgical Home: Improving the Value and Quality of Care in Total Joint Replacement. Current Reviews in Musculoskeletal Medicine, 2017. 10(3): p. 365–369. doi: 10.1007/s12178-017-9418-3 28643147 PMC5577419

[27] Agency for Healthcare Research and Quality. Patient Centered Medical Home Resource Center: Defining the PCMH. 2011 [cited 2019 April 1]; Available from: http://pcmh.ahrq.gov/portal/server.pt/community/pcmh__home/1483/PCMH_Defining%20the%20PCMH_v2.

[28] American Academy of Family Physicians, American Academy of Pediatrics, American College of Physicians, American Osteopathic Association. Joint principles of the patientcentered medical home 2007 [cited 2021 January 15]; Available from: http://www.aafp.org/dam/AAFP/documents/practice_management/pcmh/initiatives/PCMHJoint.pdf.

[29] U.S. Department of Health and Human Services Centers for Medicare and Medicaid Services. Health homes for enrollees with chronic conditions. State Medicaid Director’s Letter 10–024. 2010 [cited 2021 January 15]; Available from: http://downloads.cms.gov/cmsgov/archived-downloads/SMDL/downloads/SMD10024.pdf.

[30] U.S. Department of Health and Human Services Centers for Medicare and Medicaid Services Center for Medicare and Medicaid Innovation. State innovation models: Funding for model design and testing assistance. 2012 [cited 2021 January 15]; Available from: http://innovation.cms.gov/Files/x/StateInnovation_FOA.pdf.

[31] Cooley W.C. and McAllister J.W., Building medical homes: improvement strategies in primary care for children with special health care needs. Pediatrics, 2004. **113**(5 Suppl): p. 1499–506. 15121918

[32] Enard K.R. and Ganelin D.M., Reducing preventable emergency department utilization and costs by using community health workers as patient navigators. J Healthc Manag, 2013. 58(6): p. 412–27; discussion 428. 24400457 PMC4142498

[33] Long W.E., et al., The value of the medical home for children without special health care needs. Pediatrics, 2012. 129(1): p. 87–98. doi: 10.1542/peds.2011-1739 22184647 PMC3357044

[34] Romaire M.A., Bell J.F., and Grossman D.C., Health care use and expenditures associated with access to the medical home for children and youth. Med Care, 2012. 50(3): p. 262–9. doi: 10.1097/MLR.0b013e318244d345 22228246

[35] Strickland B.B., et al., The medical home: health care access and impact for children and youth in the United States. Pediatrics, 2011. 127(4): p. 604–11. 21402643 10.1542/peds.2009-3555

[36] Knapp C.A., et al., Factors associated with a medical home among children with Attention-Deficit Hyperactivity Disorder. Matern Child Health J, 2012. 16(9): p. 1771–8. doi: 10.1007/s10995-011-0922-6 22143465

[37] Romaire M.A., Bell J.F., and Grossman D.C., Medical home access and health care use and expenditures among children with special health care needs. Arch Pediatr Adolesc Med, 2012. 166(4): p. 323–30. doi: 10.1001/archpediatrics.2011.1154 22147757

[38] Kieckheferm G., et al., Presence and Characteristics of Medical Home and Health Services Utilization Among Children With Asthma. Journal of Pediatric Health Care, 2005. 19(5): p. 285–292. doi: 10.1016/j.pedhc.2005.05.009 16202836

[39] Diedhiou A., et al., Relationship between presence of a reported medical home and emergency department use among children with asthma. Med Care Res Rev, 2010. 67(4): p. 450–75. doi: 10.1177/1077558710367735 20442339

[40] Basu S., et al., Effects of New Funding Models for Patient-Centered Medical Homes on Primary Care Practice Finances and Services: Results of a Microsimulation Model. Ann Fam Med, 2016. 14(5): p. 404–14. doi: 10.1370/afm.1960 27621156 PMC5394379

[41] Friedberg M.W., et al., Association between participation in a multipayer medical home intervention and changes in quality, utilization, and costs of care. JAMA, 2014. 311(8): p. 815–25. doi: 10.1001/jama.2014.353 24570245 PMC6348473

[42] Al-Jahdali H., et al., Improper inhaler technique is associated with poor asthma control and frequent emergency department visits. Allergy Asthma and Clinical Immunology, 2013. **9**: p. 7. doi: 10.1186/1710-1492-9-8 23510684 PMC3605255

[43] Lynch S., et al., Toward Effective Utilization of the Pediatric Emergency Department: The Case of ADHD. Social Work in Public Health, 2016. 31(1): p. 9–18. doi: 10.1080/19371918.2015.1087909 26667350

[44] Hinde J.M., et al., Did Arkansas’ Medicaid Patient-Centered Medical Home Program Have Spillover Effects on Commercially Insured Enrollees? Inquiry-the Journal of Health Care Organization Provision and Financing, 2020. 57: p. 9. doi: 10.1177/0046958019900753 31948320 PMC6970475

[45] Swietek K.E., et al., Do Medical Homes Improve Quality of Care for Persons with Multiple Chronic Conditions? Health Services Research, 2018. 53(6): p. 4667–4681. doi: 10.1111/1475-6773.13024 30088272 PMC6232445

[46] Lindner S., et al., A Medicaid Alternative Payment Model Program In Oregon Led To Reduced Volume Of Imaging Services. Health Affairs, 2020. 39(7): p. 1194–1201. doi: 10.1377/hlthaff.2019.01656 32634361 PMC12427134

[47] Zhai S.H., et al., Multipayer Primary Care Transformation: Impact for Medicaid Managed Care Beneficiaries. American Journal of Managed Care, 2019. 25(11): p. E349. 31747240

[48] Romaire M.A., et al., Impact of Medical Homes on Expenditures and Utilization for Beneficiaries With Behavioral Health Conditions. Psychiatric Services, 2018. 69(8): p. 871–878. doi: 10.1176/appi.ps.201700433 29759058

[49] Cole E.S., et al., Patient-Centered Medical Homes In Louisiana Had Minimal Impact On Medicaid Population’s Use Of Acute Care And Costs. Health Affairs, 2015. 34(1): p. 87–94. doi: 10.1377/hlthaff.2014.0582 25561648

[50] McElligott J.T. and Summer A.P., Health Care Utilization Patterns for Young Children in Rural Counties of the I-95 Corridor of South Carolina. Journal of Rural Health, 2013. 29(2): p. 198–204. doi: 10.1111/j.1748-0361.2012.00434.x 23551650

[51] American Academy of Pediatrics. Medical Home Initiatives & Resources by State. 2020 [cited 2021 August 18]; Available from: https://medicalhomeinfo.aap.org/national-state-initiatives/Pages/National-and-State-Initiatives.aspx.

[52] United States Department of Health and Human Services. Social Risk Factors and Performance Under Medicare’s Value-Based Purchasing Program. 2014 [cited 2021 November 3]; Available from: https://aspe.hhs.gov/sites/default/files/private/pdf/253971/ASPESESRTCfull.pdf.

[53] National Academies of Sciences, E., and Medicine, Accounting for social risk factors in Medicare payment. 2017, National Academies Press: Washington (DC). 28617569

[54] US House of Representatives Document Repository. 21st Centure Cures Act. [cited 2019 August 8]; Available from: https://docs.house.gov/billsthisweek/20161128/CPRT-114-HPRT-RU00-SAHR34.pdf.

[55] National Center for Quality Assurance. Proposed Changes to Existing Measures for HEDIS®1 MY 2022: Introduction of Race and Ethnicity Stratification Into Select HEDIS Measures. 2021 [cited 2021 May 18]; Available from: https://www.ncqa.org/wp-content/uploads/2021/02/02.-Health-Equity.pdf.

[56] Institute for Families in Society. Medicaid Benefits and Services: Patients Served. 2022 [cited 2022 June 1]; Available from: https://www.schealthviz.sc.edu/patients-served.

[57] Centers for Medicare & Medicaid Services. Encounter Data Toolkit. 2013 [cited 2021 January 15]; Available from: https://www.medicaid.gov/medicaid/downloads/medicaid-encounter-data-toolkit.pdf.

[58] Lipspon D., et al., Promoting Access in Medicaid and CHIP Managed Care: A Toolkit for Ensuring Provider Network Adequacy and Service Availability. 2017, Division of Managed Care Plans, Center for Medicaid and CHIP Services, CMS. US Department of Health and Human Services: Baltimore, MD.

[59] DeVries A., et al., Impact of medical homes on quality, healthcare utilization, and costs. Am J Manag Care, 2012. 18(9): p. 534–44. 23009304

[60] Rosenthal M.B., et al., A Difference-in-Difference Analysis of Changes in Quality, Utilization and Cost Following the Colorado Multi-Payer Patient-Centered Medical Home Pilot. J Gen Intern Med, 2016. 31(3): p. 289–96. doi: 10.1007/s11606-015-3521-1 26450279 PMC4762834

[61] South Carolina Healthy Connections. Compare Health Plans. 2017 [cited 2018 November 5]; Available from: https://www.scchoices.com/Member/Step3PBECompare.aspx?frommenu=true.

[62] Pediatrics, A.A.o., The medical home. Pediatrics, 2004. 113: p. 1545–1547.15121924

[63] National Association of Pediatric Nurse Practitioners, Position Statement on Pediatric Health Care/Medical Home: Key Issues on Care Coordination, Transitions, and Leadership. Journal of Pediatric Health Care, 2014. 30(2): p. A17–A19. 27326431

[64] Billings J., Parikh N., and Mijanovish T., Emergency department use: New York City. 2000, The Commonwealth Fund, Oct 2000.

[65] Deavenport-Saman A., et al., Do Children with Autism Overutilize the Emergency Department? Examining Visit Urgency and Subsequent Hospital Admissions. Matern Child Health J, 2016. 20(2): p. 306–14. doi: 10.1007/s10995-015-1830-y 26520161

[66] Gandhi S.O. and Sabik L., Emergency department visit classification using the NYU algorithm. Am J Manag Care, 2014. 20(4): p. 315–20. 24884862

[67] Kaskie B., et al., Defining emergency department episodes by severity and intensity: A 15-year study of Medicare beneficiaries. BMC Health Serv Res, 2010. 10: p. 173. doi: 10.1186/1472-6963-10-173 20565949 PMC2903585

[68] Lin C.Y. and Lee Y.C., Appropriateness of emergency care use: a retrospective observational study based on professional versus patients’ perspectives in Taiwan. BMJ Open, 2020. 10(5): p. e033833. doi: 10.1136/bmjopen-2019-033833 32398332 PMC7223150

[69] Tuominen J., et al., NYU-EDA in modelling the effect of COVID-19 on patient volumes in a Finnish emergency department. BMC Emerg Med, 2020. 20(1): p. 97. doi: 10.1186/s12873-020-00392-1 33308165 PMC7729695

[70] Walker B.H., et al., An assessment of emergency department use among Mississippi’s Medicaid population. J Miss State Med Assoc, 2015. 56(5): p. 120–4. 26182673

[71] Ballard D.W., et al., Validation of an algorithm for categorizing the severity of hospital emergency department visits. Med Care, 2010. 48(1): p. 58–63. doi: 10.1097/MLR.0b013e3181bd49ad 19952803 PMC3881233

[72] Cook L.L., Spenceley S., and Gelber T., Clinical risk groups and patient complexity: a case study with a primary care clinic in Alberta. Health and Technology, 2019. 9(4): p. 449–461.

[73] Bell N., et al., Precision of provider licensure data for mapping member accessibility to Medicaid Managed Care provider networks. BMC Health Serv Res, 2018. 18(974). doi: 10.1186/s12913-018-3776-4 30558611 PMC6296018

[74] Bell N., et al., Community social determinants and health outcomes drive availability of patient-centered medical homes. Health Place, 2021. 67: p. 102439. doi: 10.1016/j.healthplace.2020.102439 33212394

[75] Jackson G.L., et al., Patient-Centered Medical Home A Systematic Review. Annals of Internal Medicine, 2013. 158(3): p. 169-+. 24779044 10.7326/0003-4819-158-3-201302050-00579

[76] Sinaiko A.D., et al., Synthesis Of Research On Patient-Centered Medical Homes Brings Systematic Differences Into Relief. Health Aff (Millwood), 2017. 36(3): p. 500–508. doi: 10.1377/hlthaff.2016.1235 28264952 PMC5555150

[77] Chassin M., Brook R., and Park R., Variations in the use of medical and surgical services by the Medicare population. N Engl J Med, 1986. 314: p. 285–290. doi: 10.1056/NEJM198601303140505 3510394

[78] Leape L., et al., Does inapproriate use explain small area variations in the use of health care services? JAMA, 1990. 263: p. 669–672. 2404147

[79] Newhouse J. and Garber A., Geographic variation in Medicare services. N Engl J Med, 2013. 386(16): p. 1465–1468. doi: 10.1056/NEJMp1302981 23520983

[80] Wennberg J. and Gittelsohn A., Small area variation in health care delivery. Science, 1973. 14(182): p. 1102–1108. 10.1126/science.182.4117.1102 4750608

[81] Fisher E.S., et al., The implications of regional variations in Medicare spending. Part 2: health outcomes and satisfaction with care. Ann Intern Med, 2003. 138(4): p. 288–98. doi: 10.7326/0003-4819-138-4-200302180-00007 12585826

[82] Fisher E.S., et al., The implications of regional variations in Medicare spending. Part 1: the content, quality, and accessibility of care. Ann Intern Med, 2003. 138(4): p. 273–87. doi: 10.7326/0003-4819-138-4-200302180-00006 12585825

[83] Sutherland J.M., Fisher E.S., and Skinner J.S., Getting past denial—the high cost of health care in the United States. N Engl J Med, 2009. 361(13): p. 1227–30. doi: 10.1056/NEJMp0907172 19741220

[84] Rosenbaum S., Schmucker S., and Beckerman J., Provider Networks and Access in Medicaid Managed Care: A Look at Federal and State Standards, in To the Point (blog). Commonwealth Fund, October 10, 2018.

