# Supplementary material for: Geographic proximity to primary care providers as a risk-assessment criterion for quality performance measures
Source: PLoS One. 2022 Sep 6;17(9):e0273805. doi: 10.1371/journal.pone.0273805 (PMC9447909; doi:10.1371/journal.pone.0273805)
Supplement: S1 Table — Covariates were included in the models if bivariate comparisons had p-values at or below 0.10. (DOCX) [file pone.0273805.s001.docx]

|  | **ADD Cohort** | | | | | | **MMA Cohort** | | | | | |
| --- | --- | --- | --- | --- | --- | --- | --- | --- | --- | --- | --- | --- |
|  | **Avoidable** | | | **Potentially Avoidable** | | | **Avoidable** | | | **Potentially Avoidable** | | |
| **Characteristic** | **Coeff.** | **Std. Err.** | **p value.** | **Coeff.** | **Std. Err.** | **p value** | **Coeff.** | **Std. Err.** | **p value** | **Coeff.** | **Std. Err.** | **p value** |
| Age (SD) | -0.041 | 0.019 | 0.027 | -0.050 | 0.021 | 0.018 | 0.012 | 0.005 | 0.022 | -0.024 | 0.006 | 0.000 |
| Male | -- | -- | -- | -- | -- | -- | -- | -- | -- | -- | -- | -- |
| Race/ethnicity |  |  |  |  |  |  |  |  |  |  |  |  |
| Black | 0.223 | 0.068 | 0.001 | -0.243 | 0.077 | 0.002 | 0.082 | 0.044 | 0.066 | -0.152 | 0.047 | 0.001 |
| Hispanic | 0.050 | 0.211 | 0.812 | -0.073 | 0.201 | 0.716 | 0.013 | 0.086 | 0.882 | -0.022 | 0.089 | 0.808 |
| Clinical risk groups |  |  |  |  |  |  |  |  |  |  |  |  |
| History of significant acute disease | -- | -- | -- | -- | -- | -- | -0.078 | 0.107 | 0.460 | 0.160 | 0.112 | 0.154 |
| Single minor chronic disease | -- | -- | -- | -- | -- | -- | 0.114 | 0.116 | 0.328 | 0.001 | 0.141 | 0.995 |
| Minor chronic disease in multiple organ systems | -- | -- | -- | -- | -- | -- | -0.253 | 0.281 | 0.367 | -0.177 | 0.427 | 0.678 |
| Single dominant or moderate chronic disease | -- | -- | -- | -- | -- | -- | -0.147 | 0.084 | 0.080 | 0.012 | 0.091 | 0.897 |
| Significant chronic disease in multiple organ systems | -- | -- | -- | -- | -- | -- | -0.114 | 0.093 | 0.218 | 0.042 | 0.100 | 0.674 |
| Dominant chronic disease in 3 or more organ systems | -- | -- | -- | -- | -- | -- | -1.082 | 0.616 | 0.079 | -- | -- | -- |
| Dominant and metastatic malignancies | -- | -- | -- | -- | -- | -- | -- | -- | -- | 1.178 | 0.473 | 0.013 |
| Catastrophic condition status | -- | -- | -- | -- | -- | -- | -0.476 | 0.703 | 0.498 | 1.070 | 0.290 | 0.000 |
| FFS plan | -- | -- | -- | -- | -- | -- | -0.096 | 0.097 | 0.315 | -0.087 | 0.113 | 0.442 |
| Primary care visits | -0.025 | 0.016 | 0.102 | 0.021 | 0.019 | 0.267 | 0.017 | 0.008 | 0.032 | 0.012 | 0.007 | 0.060 |
| Weekend ED visits | -- | -- | -- | -- | -- | -- | 0.078 | 0.047 | 0.039 | 0.052 | 0.0400 | 0.190 |
| Residential dwelling type |  |  |  |  |  |  |  |  |  |  |  |  |
| Suburban | -0.035 | 0.081 | 0.667 | -0.109 | 0.093 | 0.244 | -0.078 | 0.049 | 0.110 | 0.113 | 0.054 | 0.036 |
| Rural | -0.141 | 0.080 | 0.079 | -0.107 | 0.088 | 0.222 | 0.018 | 0.049 | 0.716 | 0.109 | 0.051 | 0.031 |
| Median household income | -- | -- | -- | -- | -- | -- | 0.016 | 0.141 | 0.911 | -0.049 | 0.145 | 0.738 |
| County PCMH proportion | 0.109 | 0.189 | 0.566 | 0.196 | 0.226 | 0.386 | 0.055 | 0.120 | 0.647 | 0.172 | 0.130 | 0.184 |
| PCMH attendance type |  |  |  |  |  |  |  |  |  |  |  |  |
| Attended a PCMH | -0.170 | 0.067 | 0.011 | 0.072 | 0.076 | 0.343 | 0.020 | 0.041 | 0.621 | -0.056 | 0.043 | 0.189 |
| Year |  |  |  |  |  |  |  |  |  |  |  |  |
| 2017 | -0.005 | 0.072 | 0.941 | -0.124 | 0.083 | 0.135 | 0.048 | 0.042 | 0.259 | 0.028 | 0.043 | 0.511 |
| 2018 | -0.101 | 0.082 | 0.215 | -0.070 | 0.090 | 0.434 | 0.091 | 0.047 | 0.051 | 0.023 | 0.049 | 0.643 |
