# Supplementary material for: Geographic proximity to primary care providers as a risk-assessment criterion for quality performance measures
Source: PLoS One. 2022 Sep 6;17(9):e0273805. doi: 10.1371/journal.pone.0273805 (PMC9447909; doi:10.1371/journal.pone.0273805)
Supplement: S2 Table — Covariates were included in the models if bivariate comparisons had p-values at or below 0.10. (DOCX) [file pone.0273805.s002.docx]

|  | **ADD Cohort** | | | | | | **MMA Cohort** | | | | | |
| --- | --- | --- | --- | --- | --- | --- | --- | --- | --- | --- | --- | --- |
|  | **Avoidable** | | | **Potentially Avoidable** | | | **Avoidable** | | | **Potentially Avoidable** | | |
| **Characteristic** | **Coeff.** | **Std. Err.** | **p value.** | **Coeff.** | **Std. Err.** | **p value** | **Coeff.** | **Std. Err.** | **p value** | **Coeff.** | **Std. Err.** | **p value** |
| Age (SD) | -0.042 | 0.019 | 0.025 | -0.056 | 0.021 | 0.018 | 0.011 | 0.005 | 0.041 | -0.024 | 0.006 | 0.000 |
| Male | -- | -- | -- | -- | -- | -- | -- | -- | -- | -- | -- | -- |
| Race/ethnicity |  |  |  |  |  |  |  |  |  |  |  |  |
| Black | 0.211 | 0.068 | 0.002 | -0.247 | 0.077 | 0.001 | 0.082 | 0.045 | 0.067 | -0.152 | 0.047 | 0.001 |
| Hispanic | 0.042 | 0.211 | 0.841 | -0.080 | 0.201 | 0.690 | 0.012 | 0.086 | 0.887 | 0.022 | 0.090 | 0.804 |
| Clinical risk groups |  |  |  |  |  |  |  |  |  |  |  |  |
| History of significant acute disease | -- | -- | -- | -- | -- | -- | -0.076 | 0.106 | 0.476 | 0.159 | 0.112 | 0.155 |
| Single minor chronic disease | -- | -- | -- | -- | -- | -- | 0.112 | 0.116 | 0.331 | 0.002 | 0.141 | 0.991 |
| Minor chronic disease in multiple organ systems | -- | -- | -- | -- | -- | -- | -0.264 | 0.284 | 0.359 | -0.175 | 0.427 | 0.682 |
| Single dominant or moderate chronic disease | -- | -- | -- | -- | -- | -- | -0.145 | 0.083 | 0.082 | 0.012 | 0.091 | 0.897 |
| Significant chronic disease in multiple organ systems | -- | -- | -- | -- | -- | -- | -0.105 | 0.092 | 0.255 | 0.041 | 0.101 | 0.681 |
| Dominant chronic disease in 3 or more organ systems | -- | -- | -- | -- | -- | -- | -1.092 | 0.612 | 0.075 | -- | -- | -- |
| Dominant and metastatic malignancies | -- | -- | -- | -- | -- | -- | -- | -- | -- | 1.171 | 0.472 | 0.013 |
| Catastrophic condition status | -- | -- | -- | -- | -- | -- | -0.546 | 0.661 | 0.409 | 1.081 | 0.291 | 0.000 |
| FFS plan | -- | -- | -- | -- | -- | -- |  |  |  |  |  |  |
| Primary care visits | -0.025 | 0.016 | 0.109 | 0.021 | 0.019 | 0.268 | 0.017 | 0.008 | 0.034 | 0.012 | 0.007 | 0.060 |
| Weekend ED visits | -- | -- | -- | -- | -- | -- | 0.078 | 0.037 | 0.040 | 0.052 | 0.040 | 0.190 |
| Residential dwelling type |  |  |  |  |  |  |  |  |  |  |  |  |
| Suburban | -0.029 | 0.081 | 0.717 | -0.106 | 0.093 | 0.256 | -0.071 | 0.049 | 0.148 | 0.112 | 0.054 | 0.037 |
| Rural | -0.135 | 0.080 | 0.094 | -0.107 | 0.088 | 0.236 | 0.004 | 0.049 | 0.628 | 0.109 | 0.051 | 0.032 |
| Median household income | -- | -- | -- | -- | -- | -- | 0.013 | 0.141 | 0.925 | -0.048 | 0.145 | 0.739 |
| County PCMH proportion | 0.059 | 0.191 | 0.758 | 0.179 | 0.229 | 0.436 | 0.024 | 0.121 | 0.841 | 0.176 | 0.130 | 0.174 |
| PCMH attribution type |  |  |  |  |  |  |  |  |  |  |  |  |
| un-enrolled and attended | -0.166 | 0.080 | 0.037 | 0.051 | 0.090 | 0.571 | 0.060 | 0.050 | 0.236 | -0.067 | 0.052 | 0.199 |
| enrolled and did not attend | 0.239 | 0.118 | 0.043 | 0.050 | 0.155 | 0.747 | 0.184 | 0.075 | 0.014 | -0.026 | 0.081 | 0.750 |
| enrolled and attended | -0.077 | 0.094 | 0.413 | 0.129 | 0.099 | 0.192 | 0.046 | 0.053 | 0.397 | -0.053 | 0.055 | 0.334 |
| Year |  |  |  |  |  |  |  |  |  |  |  |  |
| 2017 | -0.007 | 0.072 | 0.918 | -0.125 | 0.083 | 0.133 | 0.048 | 0.042 | 0.257 | 0.028 | 0.043 | 0.513 |
| 2018 | -0.103 | 0.082 | 0.210 | -0.070 | 0.090 | 0.435 | 0.093 | 0.047 | 0.047 | 0.023 | 0.049 | 0.645 |
