# Supplementary material for: Geographic proximity to primary care providers as a risk-assessment criterion for quality performance measures
Source: PLoS One. 2022 Sep 6;17(9):e0273805. doi: 10.1371/journal.pone.0273805 (PMC9447909; doi:10.1371/journal.pone.0273805)
Supplement: Attachment — Submitted filename: PONE-D-22-04552_R2_ReviewerResponses.docx [file pone.0273805.s004.docx]

**Reviewer #1: Summary and Overall Impression**

*Reviewer #1: Overall well written and is of great value adding to the understanding of the impact of the PCMH by assessing geographic proximity and patient outcomes.

The introduction is much more focused and reads well.

Methods again appear appropriately selected and applied.

Results appears in order well-presented.

Discussion reads well and addresses limitations and outlines future work.

Minor

Pg 11 Line 21 ..provider ? ‘over’ another.*

- Thank you for this observation. This sentence was actually from the original submission, not from revision 1. No changes were made as the sentence had been removed in our resubmission.

*Pg 14 Line 15 ..care that ? ‘is’ considered…*

- Thank you for this observation. The sentence (Page 8, Line 14 in methods section) has been amended and now reads: “…particularly care that is considered to be of higher quality”

**Reviewer #2: Summary and Overall Impression**

*Reviewer #2: 1. Rationale for the study improved with the revision but participant rationale is needed. Specifically, it is not clear why only individuals with ADD and asthma were included since the study evaluates health disparities by geographic proximity and avoidable ED visits. Examples/suggestions:*

- Thank you for mentioning this. We have amended the Methods section (page 8 of Marked Copy, Paragraph 2 and Paragraph 3) to include an additional information on the HEDIS measures and what they are and why they are important.
- Our original submission specified that these two measures (ADD, MMA) were chosen because they represent two disease conditions that are often treated in hospital EDs for care that could have been obtained by primary care providers. We have added an additional sentence (Page 8, lines 19 – 21) that re-emphasize this point.

*a. Abstract: In the background, add the need for studying ADD, Asthma; in the objective, add “potentially avoidable ED visits for children with ADD and asthma”. The conclusion (and manuscript) should state ADD and asthma since not all preexisting illnesses/chronic diseases were studied.*

- We have amended the Objective sentence in the abstract. It now reads “To examine the association between geographic proximity to primary care providers versus hospitals and risk of avoidable and potentially avoidable ED visits among children with pre-existing diagnosis of attention-deficit/hyperactivity disorder or asthma. “

*b. Introduction: (pg. 2 line 7), provide rationale for ADD and asthma (and perhaps omit homeless and diabetes) as there are many other chronic diseases that utilize EDs*

- Thank you – we have left the opening paragraph alone (as it is a framing paragraph), but have modified the 5^th^ paragraph of the introduction section (page 5, lines 6 – 9) so that the contextual information about why our study on risk adjustment included these two metrics is clear.
- Additional information was also provided in the methods section (page 8).

*2. Abstract: The conclusion is difficult to understand. Suggest 2-3 sentences stating what authors found and conclude regarding the study aim and results as well as its impact. The summary in authors’ cover letter provides much clearer conclusions.*

- Thank you for this observation. We have revised the conclusion section of the abstract. It now reads “In several health care performance evaluations, patient-centered medical homes have not been found to reduce differences in hospital utilization for conditions that are treatable in primary care settings among children with chronic illnesses. Analytical approaches that also consider geographic proximity to health care services can identify performance benefits of medical homes. Expanding risk-adjustment models to also include geographic data would benefit ongoing quality improvement initiatives.”
